# Human Milk Microbiome and Maternal Postnatal Psychosocial Distress

**DOI:** 10.3389/fmicb.2019.02333

**Published:** 2019-10-22

**Authors:** Pamela D. Browne, Marina Aparicio, Claudio Alba, Christine Hechler, Roseriet Beijers, Juan Miguel Rodríguez, Leonides Fernández, Carolina de Weerth

**Affiliations:** ^1^Department of Cognitive Neuroscience, Donders Institute for Brain, Cognition and Behaviour, Radboud University Medical Center, Nijmegen, Netherlands; ^2^Department of Nutrition and Food Science, Faculty of Veterinary Sciences, Complutense University of Madrid, Madrid, Spain; ^3^Departmental Section of Galenic Pharmacy and Food Technology, Faculty of Veterinary Sciences, Complutense University of Madrid, Madrid, Spain; ^4^Developmental Psychology, Behavioural Science Institute, Radboud University, Nijmegen, Netherlands

**Keywords:** maternal postnatal psychosocial distress, human milk, milk microbiome, bacterial diversity, time evolution

## Abstract

Human milk contains many bioactive components, including bacteria, which are transferred to the developing infant through breastfeeding. Milk bacteria appear to, amongst others, originate from the maternal gut. A mother’s postnatal psychosocial distress may alter maternal gut microbiota, which in turn may affect the bacteria present in milk. The aim of this study was to explore whether maternal postnatal psychosocial distress was related to alterations in the relative abundances of specific bacteria and to milk microbial diversity. Healthy mothers (*N* = 77; *N* = 51 with complete data) collected breast milk samples at 2, 6, and 12 weeks postpartum and filled in mood questionnaires on experienced stress, anxiety, and depressive symptoms at 6 weeks postpartum. A metataxonomic approach (16S rRNA gene sequencing (region V3 and V4) using Illumina MiSeq technology) was used to assess bacterial abundances and diversity. For the group as a whole, an increase in diversity of the milk bacterial community was observed during the first 3 months of breastfeeding (Shannon index). This general increase in diversity appears to be explained by an increase of *Lactobacillus* and other minor genera, together with a decrease in *Staphylococcus*. With respect to psychological distress and milk microbial composition, no significant differences in the relative abundance of major bacterial genera were detected between women with high (*N* = 13) and low (*N* = 13) psychosocial distress. However, progressive and distinct changes in the content of *Firmicutes*, *Proteobacteria*, and *Bacteroidetes* at the phylum level and *Acinetobacter*, *Flavobacterium*, and *Lactobacillus* at the genera level were observed in milk samples of women with low psychosocial distress. With respect to milk microbial diversity, high maternal psychosocial distress, compared to low maternal psychosocial distress, was related to significantly lower bacterial diversity in milk at 3 months post-delivery. Anxiety, stress, and depressive symptoms separately were unrelated to specific bacterial profiles. The current study suggests a potential relation between maternal psychosocial distress and milk microbiota, providing first evidence of a possible mechanism through which post-partum psychological symptoms may affect infant development and health.

## Introduction

Human milk is the most important source of nutrition for millions of infants worldwide (WHO, 2018). This highly complex biological fluid has coevolved in response to nutritional, disease, and environmental ecological pressures in human evolutionary history (Hinde and German, 2012; Allen-Blevins et al., 2015). Milk transfers many bioactive components to the developing infant, including a wide diversity of commensal bacterial genera or species (Fernández et al., 2013; Gomez-Gallego et al., 2016).

Based on the presence of bacterial DNA, previous studies indicate that milk is dominated by staphylococci (mainly by coagulase-negative staphylococci) and streptococci (*Streptococcus mitis* and *Streptococcus salivarius* groups), followed by corynebacteria, lactic acid bacteria, bifidobacteria, and propionibacteria. DNA from other microorganisms including clostridiales (*Faecalibacterium* and *Ruminococcus*) and Gram-negative bacteria (*Serratia*, *Pseudomonas*, *Sphingomonas*, *Bacteroides*, *Ralstonia*, and *Bradyrhizobiaceae*) has also been found in milk (Fernández et al., 2013; Jiménez et al., 2015; Boix-Amorós et al., 2016; Gomez-Gallego et al., 2016; Fitzstevens et al., 2017).

Sources of these milk bacteria are thought to include the infant’s and mother’s mouth and skin, breast tissue, and predominantly the maternal digestive tract (Jost et al., 2014; Rodríguez, 2014; Urbaniak et al., 2016). It is hypothesized that maternal gut microorganisms migrate to the mammary glands via an endogenous cellular route, also referred to as the bacterial entero-mammary pathway (Rodríguez, 2014). This pathway would involve the penetration of dendritic cells (DCs) and/or monocytes into the intestinal epithelium to engulf bacteria from the gut lumen. The antigen-stimulated cells, with selected gut bacteria, could then move within the mucosa-associated lymphoid tissue (MALT) to various locations, including the mammary gland (Rodríguez, 2014; de Andrés et al., 2017).

Bacteria in milk may play an important role in infant health by contributing to seeding the infant’s gut microbiota (Pannaraj et al., 2017). Given that infants are thought to ingest 1 × 10^4–8^ viable milk bacteria a day and have relatively low stomach pH and fast transit time, these bacteria are expected to be able to survive digestion and reach the infants’ intestinal tract (Jost et al., 2015). Once present in the infants’ gut, bacteria can carry out many functions, including directing the infants’ immune, and gastro-intestinal maturation (Jenmalm, 2017; Macpherson et al., 2017).

In further support of the idea of vertical transmission of bacteria from mother to infant via breastfeeding, previous studies with mother-infant pairs found that milk and infants’ fecal samples largely share a similar bacterial composition. Pannaraj et al. (2017) established that around 28% of the bacteria found in the infants’ fecal matter seems to stem from milk. Bacteria that were present in both infant fecal samples and milk included *Bifidobacterium* and *Propionibacterium* (phylum *Actinobacteria*), *Lactobacillus*, *Staphylococcus*, *Enterococcus*, *Clostridium* and *Veillonella* (phylum *Firmicutes*), *Escherichia*, *Shigella* and *Klebsiella* (phylum *Proteobacteria*), and *Bacteroides* (phylum *Bacteroidetes*) (Jost et al., 2014; Murphy et al., 2017; Pannaraj et al., 2017).

The bacterial strains present in maternal milk vary greatly between women (Fernández et al., 2013; Gomez-Gallego et al., 2016; Fitzstevens et al., 2017) and are subject to changes over time (Hunt et al., 2011; Murphy et al., 2017; Williams et al., 2017). To date, it remains largely unknown which maternal factors may explain the variation of bacteria in milk during the first months postpartum. One maternal factor that may impact the milk microbial composition is maternal postnatal psychosocial distress.

Maternal postnatal psychosocial distress refers to women experiencing symptoms of anxiety, stress, or depressive symptoms during the postnatal period. It appears to be highly prevalent, with up to 25% of women experiencing these symptoms after delivery (Priest et al., 2008; Kingston et al., 2012). Maternal postnatal psychosocial distress could potentially influence the microbial composition in milk via direct and indirect pathways. The direct pathway would involve the transfer of aberrant maternal gastrointestinal microbiota to the mammary gland through the entero-mammary pathway (Rodríguez, 2014; de Andrés et al., 2017). Studies indicate that psychosocial distress is related to altered gut microbiota in animal models and humans, including pregnant and non-pregnant women (Knowles et al., 2008; Bailey et al., 2011; Cryan and Dinan, 2012; Galley et al., 2014, 2017; Vanuytsel et al., 2014; Cerdá et al., 2016; Borgo et al., 2017; Hechler et al., 2019). Hence, milk microbial composition may be altered through transferal of altered microbiota from the maternal gut to mothers’ milk. In the indirect pathway, maternal psychosocial distress may impact the nutrient content in milk, which could in turn potentially affect the bacterial composition in milk. A clinical study showed that maternal mood was able to induce changes in the fat content in milk (Keith et al., 2012). Changes in fatty acids have been associated with variations in the milk microbiota composition. For example, higher levels of fatty acids can increase the pH of milk and consequently milk bacterial composition (Kumar et al., 2016).

To our knowledge, no studies have examined the potential association between milk microbiota composition and maternal psychosocial distress. This study takes the first steps to investigate potential associations between maternal postnatal psychosocial distress (i.e., maternal postnatal anxiety, stress, and depressive symptoms) and milk microbiota across the first 3 months postpartum in a healthy population of breastfeeding women after full term pregnancies. Specifically, we will examine milk microbial relative abundances at the phylum and genus level, and diversity of bacteria [operational taxonomic units (OTUs) and Shannon and Simpson diversity indices].

## Materials and Methods

### Participants

This project is part of the BINGO (Dutch acronym for *Biological Influences on Baby’s Health and Development*) study: a longitudinal cohort study that focuses on prenatal and early postnatal influences on infant health and development. The Ethical Committee of the Faculty of Social Sciences, Radboud University, Nijmegen, Netherlands, provided approval of the study [ECSW2014-1003-189]. Participants were recruited via the BINGO study’s website and through midwife practices, pregnancy courses, and baby stores in the region Nijmegen-Arnhem. Pregnant women, fulfilling the following criteria, were included: sufficient mastery of the Dutch language and no excessive alcohol and/or drug use (i.e., alcohol dependency and drug abuse). Both nulliparity and multiparity expectant mothers were included. The additional inclusion criteria after initial contact were no complications during pregnancy and term delivery. The inclusion criteria after birth for infants were 5-min Apgar score >7, birth weight >2,500 grams, no congenital malformations.

Eighty-eight pregnant women were willing to participate in the study, fulfilled the criteria, and signed the informed consent. Five infants born between 35 and 37 weeks of pregnancy were, otherwise, healthy and fit other inclusion criteria, and therefore were not excluded. Seventy-seven mothers (out of the 88 mothers) started breastfeeding, of which 51 mothers met the inclusion criteria for this study: no maternal antibiotic use postpartum, no breast infection postpartum, and collection of milk at all three time points (week 2, 6, and 12). Hence, breast milk samples of 51 mothers were included for analysis in this study (see Supplementary Figure S1).

Mothers filled out questionnaires on experienced stress, anxiety, and depressive symptoms at six weeks postpartum.

### Collection of Milk Samples

Mothers collected milk samples on the day after the infant reached the age of 2 weeks (time point 1) (mean age = 14.75 days, *SD* = 1.84), 6 weeks (time point 2) (mean age = 43.58 days, *SD* = 5.02), and 12 weeks (time point 3) (mean age = 85.35 days, *SD* = 2.33). The samples were collected before feeding the infant.

By hand expression, mothers collected approximately 20 mL of the first breast milk in the morning (mean time = 08:36, *SD* = 2:48). The milk was collected in small sterile cups on which mothers noted the date and time of collection. Prior to collection, mothers washed their hands, breasts, and nipples with water (unpublished results of our own lab have shown that using water to wash the breast prior to sampling yields the same results as using soap or mild antiseptics). Mothers reported whether they were or had been ill and/or taken medication in the previous week, and if so, which medication. After collection, milk samples were immediately stored in the mother’s freezers at −20°C. After the last sample was taken (approximately when the infant was 13 weeks of age), the samples were collected with a portable freezer and stored at −80°C, and subsequently sent by temperature-controlled shipment to the Complutense University of Madrid, Spain for metataxonomic analysis.

### DNA Extraction From Milk Samples

For DNA extraction, milk samples were centrifuged at 13,000 rpm for 10 min at 4°C. The pellets were washed with TE buffer (10 mM Tris–HCl, 50 mM EDTA, pH 8). Then, the samples were mechanically lysed using the FastPrep-24 (MP Biomedicals, Solon, OH, United States) and glass beads matrix tubes (2 cycles × 30 s, speed 6), keeping the tubes on ice between cycles. The samples were centrifuged at 13,000 rpm for 1 min at 4°C and the supernatants were incubated with 200 μL of an enzyme mixture containing lysozyme (10 mg/mL), mutanolysin (10,000 U/mL), and lysostaphin (4,000 U/mL) at 37°C for 90 min. The samples were further incubated at 56°C for 30 min with proteinase K (250 μg/mL) to eliminate the protein fraction from the supernatant. Finally, the DNA was extracted using the QIAamp DNA Stool Kit (Qiagen, Hilden, Germany). Extracted DNA was eluted in 22 μL of nuclease-free water and stored at −20°C until further analysis. Purity and concentration of each extracted DNA was estimated using a NanoDrop 1000 spectrophotometer (NanoDrop Technologies, Inc., Rockland, ME, United States).

### PCR Amplification and Sequencing

A dual-barcoded 2-step PCR reaction was conducted to amplify a fragment of the V3–V4 hypervariable region of the bacterial 16S ribosomal RNA (rRNA) genes. Equimolar concentrations of the universal primers S-D-Bact-0341-b-S-17 (5′-CCTACGGGNGGCWGCAG-3′) and S-D-Bact-0785-a-A-21 (5′- GACTACHVGGGTATCTAATCC-3′) were used as previously described (Klindworth et al., 2013), generating amplicons of approximately 464 bp from the V3-V4 hypervariable region. In the second PCR-reaction, barcodes used for Illumina sequencing were appended to 3′ and 5′ terminal ends of the PCR amplicons to allow for the separation of forward and reverse sequences. The 2100 Bioanalyzer system (Agilent, Santa Clara, CA, United States) was used to determine the DNA concentration of each sample.

Barcoded PCR products from all samples were pooled at approximately equimolar DNA concentration and run on a preparative agarose gel. The band having the correct size was excised and purified using a QIAEX II Gel Extraction Kit (Qiagen) and then quantified with PicoGreen (BMG Labtech, Jena, Germany). Finally, one aliquot of pooled, purified, and barcoded DNA amplicons was sequenced using the Illumina MiSeq pair-end protocol (Illumina Inc., San Diego, CA, United States) according to the manufacturer’s protocols at the facilities of Parque Científico de Madrid (Tres Cantos, Spain). Samples of negative extraction controls and nuclease-free water used in PCR reactions were also run and included in each PCR reaction. Electrophoresis (1% agarose gel) showed no visible band in these negative controls and, therefore, these PCR products were not sequenced.

The amplified fragments, the forward and reverse Illumina reads, were merged into single reads using SeqPrep^1^ allowing a maximum of 0.5 mismatched bases. The resulting high-quality reads were assembled and classified taxonomically into OTUs by comparison with the Greengenes database (version 13_8) (DeSantis et al., 2006) using a Bayesian classification method and a level of similarity of at least 97%.

### Postnatal Psychosocial Distress

#### Postnatal Stress

Postnatal stress was assessed using the Alledaagse Problemen Lijst (Everyday Problem Checklist; APL; Vingerhoets et al., 1989). The APL assessed the occurrence of and intensity of daily hassles. The questionnaire consisted of 49 items and mothers indicated whether a daily hassle had occurred in the past 2 months and how much it bothered them on a 4-point Likert scale. The scale ranged from 0 (“I do not mind at all”) to 3 (“I do mind a lot”).

To calculate the mean intensity rating of daily hassles, the sum of total (negative) valence was divided by the frequency of the events. Higher values indicate more experienced negativity. The internal consistency in this sample was sufficient with a Cronbach’s α of 0.84.

#### Postnatal General Anxiety

Postnatal general anxiety was measured with a Dutch translation of the State Trait Anxiety Inventory (STAI-S; Spielberger et al., 1983). This STAI-State measure consisted of 20 items for which participants indicated whether they experienced feelings of anxiety at the present moment on a 4-point scale ranging from 1 (“not at all”) to 4 (“very much”). Scores were calculated by summing up the scores of the 20 items, with higher scores indicating more general feelings of anxiety. Internal consistency in this sample was excellent with Cronbach’s α equal to 0.90.

#### Postnatal Depressive Symptoms

The Edinburgh Postnatal Depression Scale was used to measure postnatal depressive symptoms (EPDS; Cox et al., 1987). The EPDS consisted of 10 items related to experienced feelings of depression. Participants indicated whether they experienced feelings of depression in the past 7 days on a 4-point scale with a range of 0 to 3 in seriousness of symptoms. A sum score of the 10 items was computed, with higher scores indicating more feelings of depression. The internal consistency in this sample was good with Cronbach’s α equal to 0.87.

#### Operationalization of Maternal Psychosocial Distress

Maternal postnatal distress can be regarded as a multidimensional concept of different symptoms: women experiencing maternal psychosocial distress often report more than one symptom of distress (i.e., symptoms of anxiety, stress, and/or depression) (Heron et al., 2004; Nast et al., 2013). However, there is a lack of a validated questionnaire to measure maternal postnatal distress in a multidimensional fashion. For this reason, and also in order to reduce the number of analyses, the APL, STAI-S, and EPDS were used together to set up subgroups for analysis. Two subgroups of women were extracted from the sample: the women with the highest (group H) and lowest (group L) scores on psychosocial distress (Zijlmans et al., 2015). To this end, we computed a median score for each of the three psychosocial distress variables. Women scoring above the median on all three variables were categorized as the “high maternal postnatal psychosocial distress group” (group H) (*N* = 13, 25%), while mothers scoring below the median on all three variables were categorized as “low maternal postnatal psychosocial distress group” (group L) (*N* = 13, 25%).

### Statistical Analysis

All datasets were tested for normality using Shapiro-Wilk’s tests. Continuous variables are reported as means and 95% confidence interval (CI) or standard deviation (SD) or medians and interquartile range (IQR), as appropriate. Categorical variables are given as percentages.

Bioinformatics’ analyses to process 16S rRNA amplicon data were conducted using QIIME version 1.9.1 (Caporaso et al., 2010). A table of OTU counts per sample was generated, and bacterial taxa abundances were normalized to the total number of sequences in each sample. Then, sequences were rarified to the minimum number sequences common to all the samples before analysis of alpha and beta diversities (Supplementary Figure S2). Alpha diversity was studied with the Shannon and Simpson diversity indices (Haegeman et al., 2013). Beta diversity studies were performed using the principal coordinates analysis (PCoA) to plot patterns of bacterial community diversity through a distance matrix containing a dissimilarity value for each pairwise sample comparison. Quantitative (relative abundance) and qualitative (presence/absence) analyses were performed with the Bray-Curtis index and binary Jaccard index, respectively. PERMANOVA analysis with 999 permutations were used to reveal statistically significant differences.

Due to possible autocorrelation between samples taken from the same participant, when appropriate, a repeated measures experimental design was employed to explore longitudinal differences in relative abundances of milk microbiota at phylum and genus level and diversity indexes (either using repeated measures ANOVA or Friedman tests). Otherwise, differences between groups were assessed using one-way or multifactorial ANOVA or Kruskal-Wallis tests. Multiple comparisons were adjusted with Bonferroni correction. Appropriate *post hoc* analysis were performed to identify which groups were significantly different. Retrospective statistical power calculation was done using GPower v 3.1 (Faul et al., 2009). Statistical analysis and plotting was performed in the R environment using R version 3.5.1 (R Core Team, 2013)^2^ with library *ggplot2* (Wickham, 2016). Internal consistency (Cronbach’s alpha) was measured using the *psych* package in R (Revelle, 2018). Differences were considered statistically significant at *p* < 0.05.

## Results

### Descriptives

The demographic characteristics of mothers participating in the study are summarized in Table 1. Mean (SD) maternal age was 32.0 (3.7) years, ranging from 25 to 40 years. Most participants come from educationally advantaged backgrounds. The infants were born at a mean (SD) gestational age of 39.73 (1.66) weeks, with approximately the same number of boys (*n* = 25), and girls (*n* = 26) (Table 1). Women included in this study scored relatively low on experienced stress, anxiety, and depressive symptoms compared to clinical population. For example, in the total group of women, median score of depressive symptoms was 5, while a score of 10 of more on the EPDS questionnaire suggest that mild or major depression may be present (Bergink et al., 2011). Similarly, the median score on anxiety was 27, however, a score of 40 or more on STAI indicates increased levels of prenatal anxiety (Grant et al., 2008).

**TABLE 1:** Participant information for the group as a whole, as well as for the low psychosocial stress (L) and high psychosocial stress (H) groups separately.

	**Total (*N* = 51)**	**Group L (*n* = 13)**	**Group H (*n* = 13)**	***p*-value**

**Maternal characteristics**				

**Maternal age (years)**				
Mean (SD)	32.04 (3.70)	30.50 (3.37)	32.69 (4.09)	0.159^d^
Range	26.00–40.00	25.00–36.00	28.00–40.00	
**Educational background (*n*)**				
*Middle level vocational education*	8	3	1	0.621^e^
*Higher professional education*	15	3	2	
*Higher university education*	26	7	9	
*Other*	2	0	1	
**Psychosocial distress [median (IQR)]**				
*Postnatal stress*^a^	2.14 (1.83–2.50)	1.86 (1.60–2.00)	2.50 (2.40–2.67)	<0.001^f^
*Postnatal general anxiety*^b^	27.0 (23.0–32.0)	24 (22–25)	38.0 (32.0–43.0)	<0.001^f^
*Postnatal depressive symptoms*^c^	5.0 (3.0–7.0)	3.0 (0.0–6.0)	10.0 (8.0–11.0)	<0.001^f^

**Infant characteristics**				
**Infant sex (n)**				
*Boy*	25	7	6	0.699^g^
*Girl*	26	6	7	
**Gestational age at birth (weeks)**				
Mean (SD)	39.73 (1.66)	39.95 (1.38)	39.93 (1.28)	0.966^d^
Range	35.57–42.14	36.71–41.71	37.00–41.71	

*^*a*^Alledaagse Problemen Lijst (Everyday Problem Checklist; EPL; Vingerhoets et al., 1989); scale: 0–342. ^*b*^State Trait Anxiety Inventory (STAI-S; Spielberger et al., 1983); scale: 20–80. ^*c*^Edinburgh Postnatal Depression Scale (EPDS; Cox et al., 1987); scale: 0–30. ^*d*^One-way ANOVA test was used to determine differences in gestational age at birth between groups L and H. ^*e*^Fisher exact probability test was used to evaluate differences in mother’s educational background between groups L and H. ^*f*^Kruskal-Wallis tests were used to evaluate differences in the postnatal stress, general anxiety and depressive symptoms between groups L and H. ^*g*^Chi-square test was used to evaluate differences in infant sex between groups L and H.*

### Preliminary Analysis: Sequencing Summary

A total of 152 human milk samples collected from 51 individuals were sequenced in the V3-V4 rRNA hypervariable region resulting in 253,948 usable reads (mean = 1670, *SD* = 577 reads/sample, ranging from 361 to 3748) and 718 OTUs (median = 41.5, IQR = 29.0–59.5 OTUs/sample) according to 97% species similarity (Supplementary Table S1).

Apart from OTUs belonging to domain Bacteria (median = 98.20%, IQR = 96.67–99.21%), some OTUs belonging to domain Archaea were identified in 20 (13%) samples in very low amounts (median = 0.12, IQR = 0.08–0.21) (Supplementary Table S1). All samples had a low number (median = 1.78%, IQR = 0.75–3.21%) of unclassified OTUs (Supplementary Table S1). Assembled OTUs belonged to known 22 phyla, 48 classes, 86 orders, 169 families, and 335 genera.

### Preliminary Microbial Analysis at the Phylum Level: Detection Frequencies, Relative Abundance, and Changes Across Time

**FIGURE 1:**
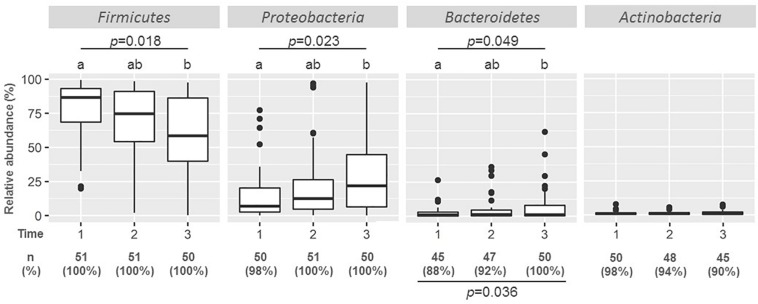
Boxplots of the relative abundance of main phyla in milk samples taken at weeks 2 (*n* = 51), 6 (*n* = 51), and 12 (*n* = 50). Friedman tests were used to evaluate differences in relative abundances along time, and the *p*-values are shown under the phylum name; different caption letters mean statistical differences when the *post hoc* pairwise comparison Nemenyi test was done. The frequency of detection [number (%) of samples in which phylum assignments were detected] is also shown at the bottom of the graph, Fischer exact probability tests were used to evaluate differences in the detection of the phylum along time, and the *p*-values are shown under the frequencies of detection.

The most frequent and abundant phylum was *Firmicutes* which was present in all the samples (*N* = 152) (Figure 1). At week 2, *Firmicutes* accounted for 86.51% of the OTUs, but its content decreased to 74.54% of the OTUs at week 6, and a further decrease to 58.51% was registered at week 12 (*p* = 0.018; Friedman test).

*Proteobacteria* was the second most frequent phylum, being present in almost all the samples (Figure 1). It was also the second most abundant phylum, although its relative abundance was notably lower than that of *Firmicutes* with a median (IQR) relative abundance of 7.87% (2.60–20.34%) at week 2. Contrary to *Firmicutes*, the relative abundance of *Proteobacteria* increased with time, being 12.35 and 21.79% at weeks 6 and 12, respectively. This change was also statistically significant (*p* = 0.023; Friedman test).

Other Gram-negative phyla present in most of the samples were *Actinobacteria* and *Bacteroidetes* detected in 98 and 88% of the samples at week 2, respectively (Figure 1). While the number of samples where *Bacteroidetes* was detected increased significantly along time (*p* = 0.036, Fisher exact probability test) from 88% at week 2 and 92% at week 6, to 100% at week 12, that of *Actinobacteria* decreased from 98% at week 2 to 90% at week 12, although this change was not statistically significant. The relative abundance of *Bacteroidetes* also increased with time (*p* = 0.049; Friedman test). In contrast to *Firmicutes* and *Proteobacteria*, the relative abundance of *Bacteroidetes* and *Actinobacteria* was very low (median value < 2.5% of total OTUs).

Most of the samples contained OTUs that were annotated to minor phyla having very low relative abundance (median value < 1% of the total) (Supplementary Table S2). Unclassified OTUs were present in all samples, and their median relative abundance varied between 1.87% (week 2) and 1.73% (week 12) (Supplementary Table S2).

### Preliminary Microbial Analysis at the Genus Level: Detection Frequencies, Relative Abundance, and Changes Across Time

**FIGURE 2:**
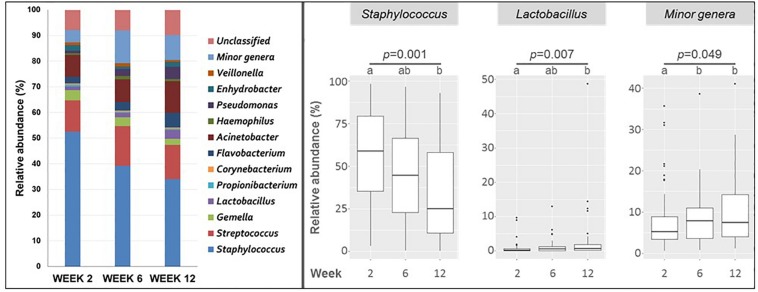
Left, mean relative abundance of major bacterial genera in milk samples collected at weeks 2 (*n* = 51), 6 (*n* = 51), and 12 (*n* = 50). Minor genera covers bacterial genera with a relative abundance of <0.1%. Right, boxplots showing the change in relative abundance of bacterial genera in milk samples taken at weeks 2, 6, and 12. Only changes having a statistical significant change (Friedman tests) are shown. The *p*-values are shown under the genus name; different caption letters mean statistical differences when the *post hoc* pairwise comparison Nemenyi test was done.

At the genus level, *Staphylococcus* was detected in all samples and was the most abundant genus (Figure 2). In milk samples collected at week 2, the median (IQR) value of the total OTUs belonging to *Staphylococcus* was 58.71% (35.33–79.60%) of the total, but its abundance decreased significantly over time (44.65% at week 6 and 24.81% at week 12; *p* = 0.001).

*Streptococcus* was also detected in all samples at week 2, and in most of the samples (96% and 94% of total) collected at weeks 6 and 12, respectively (Supplementary Table S3). The median (IQR) value of the total OTUs belonging to *Streptococcus* [8.02% (2.50–20.94%)] was much lower than that of *Staphylococcus* at week 2 and did not change significantly during the study [10.72% (2.63–27.46%) at week 6 and 7.46% (1.94–20.31%) at week 12].

Other genera belonging to *Firmicutes*, such as *Corynebacterium*, *Gemella*, *Propionibacterium*, and *Lactobacillus*, were present in a high number of samples (in 80%, 76%, 67% and 67% of the samples, respectively) at week 2 (Figure 2). Similar frequencies of detection were registered for weeks 6 and 12 (Supplementary Table S3). However, the number of OTUs of these four genera represented only a small proportion of the total, ranging from a median value of 3.20% for *Gemella* to 0.21% for *Corynebacterium* at week 2. Small variations were observed for samples taken at weeks 6 and 12. *Lactobacillus* was significantly more abundant in samples collected at weeks 6 and 12 than in initial samples taken at week 2 (*p* = 0.007) (Figure 2).

*Acinetobacter* was the genus of the *Proteobacteria* phylum most frequently detected in milk samples (it was present in 71% of the samples taken at week 2) (Figure 2). Other genera of the *Proteobacteria* phylum such as *Haemophilus*, *Pseudomonas*, and *Enhydrobacter* were also present in approximately the same number of samples (between 69% and 61%) at week 2. The frequency of detection was similar in samples taken in weeks 6 and 12. However, the median relative abundance of any of these genera was lower than 1% (Supplementary Table S3).

The genus *Flavobacterium* was detected in more than half of the samples (55%) collected at week 2, with a median (IQR) relative abundance of 1.72% (0.36–3.79%). Similar detection frequencies and median relative abundances were registered in samples taken at weeks 6 and 12 (Supplementary Table S3).

### Preliminary Analysis: Bacterial Abundance and Diversity

Richness (observed OTU’s), Shannon-Wiener index, and Simpson’s index were calculated to describe the diversity of the milk microbial community (Supplementary Table S4). Individual samples had a median (IQR) number of different OTUs of 38 (26–49) and ranged from 10 to 163 at week 2. Globally, milk samples were characterized by low numbers of highly frequent OTUs. OTU richness of milk samples within each participant did not change with time (*p* = 0.058, Friedman test) (Supplementary Table S4).

**FIGURE 3:**
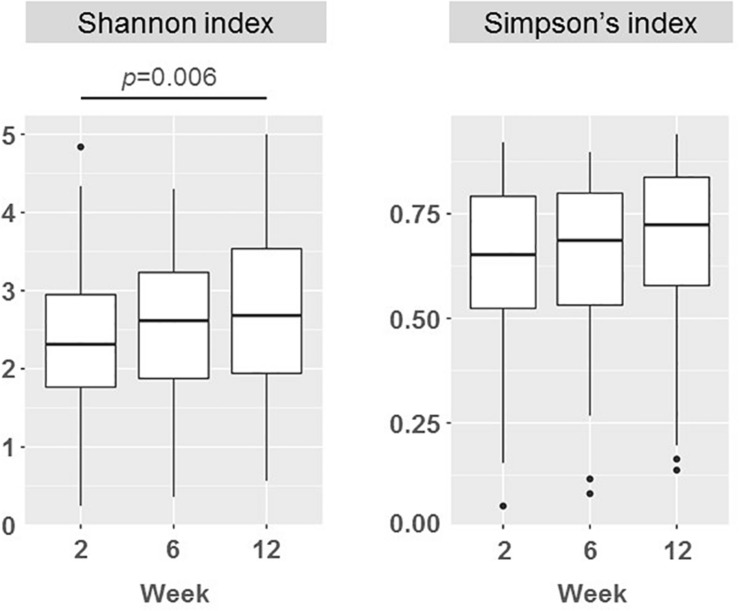
Boxplots showing the biodiversity measured as the Shannon and the Simpson indices in milk samples in weeks 2 (*n* = 51), 6 (*n* = 51), and 12 (*n* = 50). Repeated measures ANOVA and Friedman tests were used to evaluate the change in Shannon and Simpson indices, respectively. *p*-values show significantly statistical differences.

There was a wide variation in bacterial diversity among milk samples (Figure 3). The mean (95% CI) value of the calculated Shannon index in samples taken at week 2 was 2.38 (2.18, 2.58) but it ranged from 0.25 to 4.84. The microbial biodiversity in milk samples increased slightly with time, reaching a mean (95% CI) value of 2.68 (2.48–2.89) for the Shannon index at week 12. This change (mean rise of 0.30 in the Shannon index) was statistically significant when it was evaluated for the samples from each participant (*F* = 1.82, *p* = 0.006; repeated measures ANOVA). However, the Shannon index, that it is more sensitive to evenness because it gives more weight to rare than to abundant OTUs, may be underestimated due to incomplete coverage. The Simpson’s index, which is weighted more toward dominant OTUs, did not change over time (*p* = 0.923; Friedman test) (Figure 3).

### Main Analysis: Psychosocial Distress and Milk Microbiota

**FIGURE 4:**
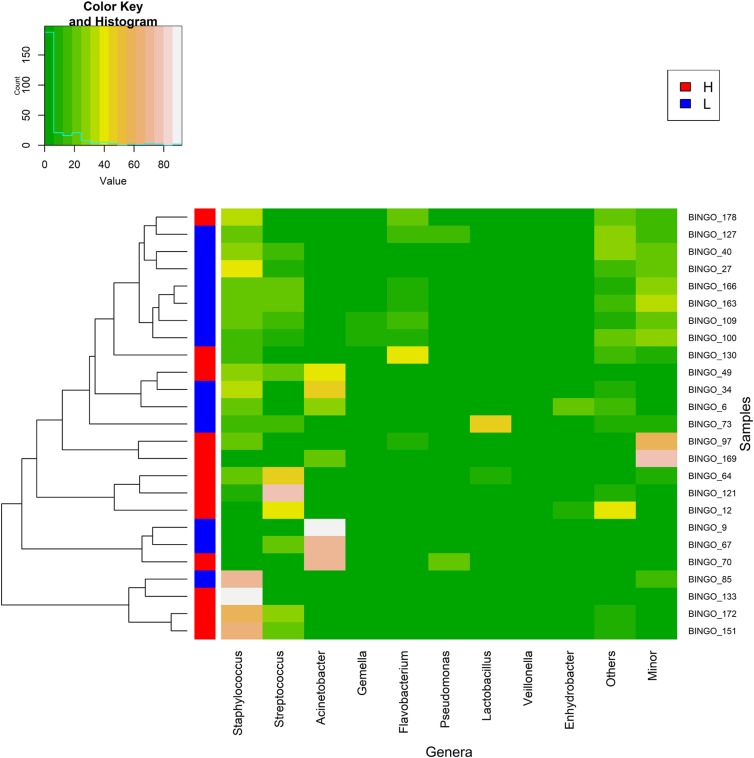
Heat map showing the relative abundance of selected most abundant bacterial genera (*x* axis) detected in milk samples (*y* axis; each row represents one milk sample) from women with low (L, blue; *n* = 13) and high (H, red; *n* = 12) postnatal psychosocial distress at week 12. The relative abundance of OTUs within each sample is indicated by the color of the scale ranging from white (high relative abundance) to dark green (low relative abundance) as indicated in the scale shown at the left upper corner. Dendogram linkages are based upon relative abundance of the genus within the samples using *hclust* as the clustering algorithm. The column between the dendogram of the milk samples and the individual values of the relative abundance of bacterial genera indicates the level (L, low in red; H, high in blue) of postnatal psychosocial distress.

To evaluate the association between maternal psychosocial distress and milk microbiota subgroups of women with high (group H, *n* = 13) and low (group L, *n* = 13) psychosocial distress were extracted from the data. There were no differences in maternal characteristics, gestational age at birth, infant birth weight, delivery mode, place of birth and the proportion of male vs. female infants between the two groups (Table 1). As expected, women in the high psychosocial stress group (H) scored significantly higher on all three measures than the women in the low psychosocial stress group (L) (Table 1). Globally, the relative abundances of major bacterial genera in milk samples provided by women with the lowest (group L) and highest (group H) psychosocial distress at week 12 did not allow to form distinct clusters (Figure 4). However, when changes in the relative abundance of main bacterial phyla and genera in the samples provided by each participant in groups L and H were analyzed, significant changes were found over time (Tables 2, 3). The individual decrease in the median relative abundances of *Firmicutes* and the increase in those of *Proteobacteria* and *Bacteroidetes* was statistically significant (*p* = 0.012, *p* = 0.004, and *p* = 0.041, respectively) for women in group L, but not for women in group H (Table 2). Some changes were also observed at the genus level. For women in group L, the individual relative abundance of *Acinetobacter* (*p* = 0.044), *Flavobacterium* (*p* = 0.004), and *Lactobacillus* (*p* = 0.009) increased significantly during the first postnatal months. In contrast, relative abundances of the main bacterial genera did not change over time in group H, except for *Staphylococcus* (Table 3).

**TABLE 2:** Individual change in the relative abundance (% of total) of operational taxonomic units at phylum level in milk samples from participants in groups L and H at weeks 2, 6, and 12 after delivery.

		**Week 2**	**Week 6**	**Week 12**	
	**Phylum**	**Median (IQR)**	**Median (IQR)**	**Median (IQR)**	***p*-valueⱡ**
GROUP L	*Firmicutes*	91.14 (85.00-92.66)a	78.43 (70.13-82.78)ab	49.15 (29.86-56.44)b	0.012
(*n* = 13)	*Proteobacteria*	4.43 (2.60-6.90)a	11.93 (6.18-19.82)ab	29.46 (21.68-48.23)b	0.004
	*Actinobacteria*	0.87 (0.34-2.70)	0.64 (0.26-2.58)	1.90 (0.74-2.59)	0.162
	*Bacteroidetes*	0.66 (0.34-2.05)	1.58 (0.86-4.48)	4.26 (1.59-11.78)	0.041
GROUP H	*Firmicutes*	82.39 (69.10-89.58)	84.92 (55.78-92.73)	52.10 (36.53-88.78)	0.920
(*n* = 12)	*Proteobacteria*	10.96 (3.74-20.27)	6.07 (2.97-19.48)	15.05 (2.35-46.72)	0.779
	*Actinobacteria*	0.87 (0.50-1.29)	0.59 (0.37-1.04)	0.76 (0.24-1.20)	0.938
	*Bacteroidetes*	1.26 (0.72-3.22)	1.06 (0.28-3.60)	0.96 (0.35-4.61)	0.920

*
ⱡFriedman tests were used to evaluate differences in the relative abundance of the genera along time within individuals. Different caption letters mean statistical differences when the *post hoc* pairwise comparison Bonferroni test was done.*

**TABLE 3:** Individual change in the relative abundance (% of total) of operational taxonomic units at genus level in milk samples from participants in groups L and H at weeks 2, 6 and 12 after delivery.

		**Week 2**	**Week 6**	**Week 12**	
	**Genus**	**Median (IQR)**	**Median (IQR)**	**Median (IQR)**	***p*-valueⱡ**
GROUP L	*Staphylococcus*	59.86 (56.77–79.05)a	45.60 (23.24–60.02)ab	20.29 (17.22–25.12)b	0.018
(*n* = 13)	*Streptococcus*	10.58 (6.97–20.94)	7.42 (4.15–32.59)	10.89 (4.74–14.94)	0.926
	*Gemella*	1.50 (0.20–5.53)	1.47 (0.88–2.37)	1.11 (0.11–2.77)	0.583
	*Acinetobacter*	0.17 (0.00–1.12)	0.13 (0.00–1.94)	1.77 (0.53–30.47)	0.044
	*Pseudomonas*	0.06 (0.00–0.28)	0.36 (0.00–1.19)	0.28 (0.00–1.05)	0.439
	*Lactobacillus*	0.20 (0.00–0.56)a	0.62 (0.22–1.41)ab	1.39 (0.29–3.47)b	0.009
	*Veillonella*	0.06 (0.00–0.10)	0.17 (0.00–0.27)	0.22 (0.11–0.33)	0.210
	*Enhydrobacter*	0.10 (0.00–0.20)	0.14 (0.00–0.33)	0.55 (0.15–0.74)	0.059
	*Flavobacterium*	0.00 (0.00–0.36)a	0.03 (0.00–0.77)ab	0.49 (0.00–10.01)b	0.004
GROUP H	*Staphylococcus*	48.75 (27.54–69.90)	40.88 (7.43–79.53)	21.88 (6.60–45.94)	0.046
(*n* = 12)	*Streptococcus*	10.50 (1.43–21.71)	8.73 (5.61–39.17)	11.88 (2.49–32.68)	0.920
	*Gemella*	2.74 (0.23–9.15)	0.86 (0.34–3.29)	0.20 (0.00–0.45)	0.094
	*Acinetobacter*	0.51 (0.02–1.89)	0.80 (0.27–1.70)	0.33 (0.03–10.98)	0.203
	*Pseudomonas*	0.19 (0.00–0.46)	0.34 (0.00–0.44)	0.11 (0.00–1.35)	0.614
	*Lactobacillus*	0.33 (0.00–0.55)	0.13 (0.00–0.72)	0.19 (0.03–0.93)	0.600
	*Veillonella*	0.15 (0.00–1.68)	0.27 (0.02–2.59)	0.00 (0.00–0.31)	0.139
	*Enhydrobacter*	0.07 (0.00–0.37)	0.03 (0.00–0.50)	0.10 (0.00–0.56)	0.558
	*Flavobacterium*	1.04 (0.00–2.98)	0.21 (0.00–1.55)	0.58 (0.03–5.14)	0.924

*
ⱡFriedman tests were used to evaluate differences in the relative abundance of the genera along time within individuals. Different caption letters mean statistical differences after the *post hoc* pairwise comparison Bonferroni test was done.*

**FIGURE 5:**
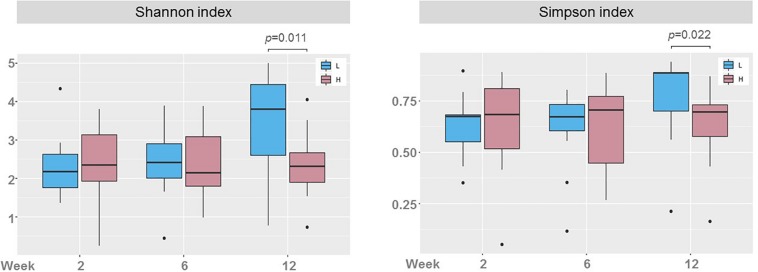
Boxplots showing the biodiversity, measured as the Shannon and the Simpson indices, in milk samples from women with low (L, blue; *n* = 13) and high (H, red; *n* = 13, except at week 12, where *n* = 12) postnatal psychosocial distress across time (weeks 2, 6, and 12). One-way ANOVA and Kruskal-Wallis tests were used to evaluate differences in Shannon and Simpson indices, respectively, between samples of groups L and H at each sampling time (*p*-values show statistically significant differences).

The Shannon diversity indexes in milk samples of women in groups H and L were compared (Figure 5). Statistically significant differences concerning microbial diversity were found between the two groups, but only in samples taken at week 12: the mean (95% CI) Shannon diversity index was 3.47 (3.03, 3.91) in group L and 2.37 (1.91, 2.83) in group H (*p* = 0.011; one-way ANOVA) and the median (IQR) Simpson diversity index was 0.89 (0.70–0.89) for group L and 0.70 (0.58–0.73) for group H (*p* = 0.022; Kruskal-Wallis test). The retrospective computed achieved power for the one-way ANOVA test to evaluate the differences found in Shannon indices between milk samples of groups L and H at week 12 was 0.798 (calculated effect size d = 1.023). For the Kruskal-Wallis test to evaluate the differences found in Simpson diversity indices, the power was 0.559 (calculated effect size *d* = 0.749). When comparing groups L and H, there was a different change in bacterial diversity over time (Figure 5). While the Shannon and Simpson indices did not change over time in samples of women in group H, there was an increase in alfa diversity in samples of women in group L, and the change was statistically significant (*F* = 6.52, *p* = 0.004, one-way ANOVA for Shannon index; and *p* = 0.007, Kruskal-Wallis test for Simpson index). The change in biodiversity was specially marked between weeks 6 and 12.

Exploratory analyses looking at the relation between milk microbiota (i.e., relative abundances and diversity) and anxiety, stress, and depressive symptoms separately, showed the same patterns of bacterial profiles and diversity than those of the aggregate measure of postnatal psychosocial distress (results not presented).

**FIGURE 6:**
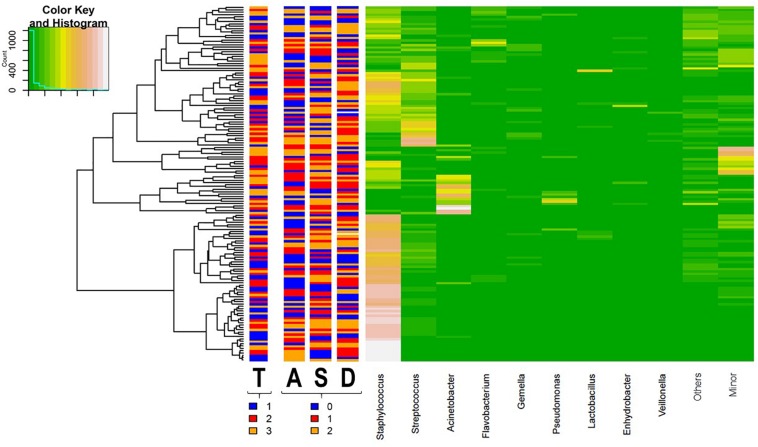
Heat map showing the relative abundance of selected most abundant bacterial genera (*x* axis) detected across all milk samples (*y* axis; each row represents one milk sample; *n* = 152). The relative abundance of OTUs within each sample is indicated by the color of the scale ranging from white (high relative abundance) to green (low relative abundance) as indicated in the scale shown at the left upper corner. Dendogram linkages are based upon relative abundance of the genus within the samples and *hclust* was used as the clustering algorithm. The columns between the dendogram of the milk samples and the individual values of the relative abundance of bacterial genera indicates the sampling time (T) (1, week 2 in blue; 2, week 6 in red; and 3, week 12 in orange) and the score level (0, low in blue; 1, medium in red; and 2, high in orange; samples for which the score level of the variable was unknown are colored in white) of postnatal psychosocial distress variables (A for anxiety, S for stress, and D for depressive symptoms).

The distribution pattern of the bacterial genera present in all samples analyzed is shown in a heat map (Figure 6). Samples clustered in two groups or branches that were characterized by different taxonomic composition. One group, comprising 63 (41%) samples, was characterized by high abundance of *Staphylococcus*, while a mixture of different patterns was found either having predominance of *Acinetobacter* (a), a mixture of minor genera (b) or *Streptococcus* (c), or being a mixture of almost exclusively *Staphylococcus* and *Streptococcus* (d) or a mixture of roughly similar amounts of *Staphylococcus*, *Streptococcus, Flavobacterium, Gemella*, and other less frequent or minor bacterial genera (e) in the other group. This clustering did not correspond with homogeneous groups of samples including a particular level of any variable of psychosocial distress or a sampling time, although samples taken at the beginning of the study (week 2) showed a trend to fall in the cluster with a high abundance of *Staphylococcus* (Figure 6).

## Discussion

The aim of this study was to examine the potential association between maternal psychosocial distress (i.e., maternal postnatal anxiety, stress, and depressive symptoms) and milk microbiota in a healthy population of breastfeeding women after full term pregnancies. Although studies on variations and evolution over time of milk bacterial composition are still scarce, some factors, such as gestational age, geographic location, antibiotic usage, or maternal diet, are known to influence the composition of milk microbiota (Khodayar-Pardo et al., 2014; Soto et al., 2014; Toscano et al., 2017; Williams et al., 2017; Biagi et al., 2018; Meehan et al., 2018). In contrast, to date, maternal psychosocial distress had not been addressed as a potential factor, which may influence the milk bacterial composition (i.e., relative abundance and diversity).

Consistent with results of previous studies using different culture-dependent and -independent approaches, milk samples contained of a wide range of bacterial genera, with *Staphylococcus* and *Streptococcus* being the most abundant ones (Jost et al., 2013; Jiménez et al., 2015; Boix-Amorós et al., 2016; Gomez-Gallego et al., 2016; Urbaniak et al., 2016; Fitzstevens et al., 2017; Murphy et al., 2017; Williams et al., 2017). In addition, our results suggest that psychosocial distress is related to some changes in the relative abundance of milk bacteria and to lower milk bacterial diversity. While microbial diversity increased in women with low psychosocial distress from week 2 to week 12, diversity remained relatively stable in women with high psychosocial distress during this period; the difference between women with low and high psychosocial distress for diversity became significant at 3 months postpartum.

The increase in bacterial diversity among women with low psychosocial distress was mostly driven by a decrease in the relative abundance of the main bacterial genera (*Staphylococcus*) and the increase of some minority genera (*Lactobacillus*, *Acinetobacter*, and *Flavobacterium*). Previous studies report a similar decrease in the relative abundances of *Staphyloccus* in both human milk and breast-fed infants’ feces during the first 4 weeks and 6 months post-delivery (Adlerberth et al., 2006; Jiménez et al., 2008; Cabrera-Rubio et al., 2012). Although *Lactobacillus*, *Acinetobacter*, and *Flavobacterium* have been found to be among the core milk microbiome genera in different studies, there are no previous reports describing changes in relative abundance of these genera during the first months postpartum (Jiménez et al., 2015; Boix-Amorós et al., 2016; Sakwinska et al., 2016; Murphy et al., 2017; Pannaraj et al., 2017).

Given that some commensal bacteria from the maternal digestive tract may be transferred to the mammary gland, the difference in the bacterial diversity at 3 months post-delivery of the milk microbiota may be, at least in part, driven by changes in the maternal gut microbiota (Perez et al., 2007; Rodríguez, 2014; de Andrés et al., 2017). Psychosocial distress may have altered maternal gut microbiota, and hence milk microbiota, through two axes: the microbiota-gut-brain axis and the hypothalamic-pituitary-adrenal (HPA) axis. The microbiota-gut-brain axis is a bidirectional signaling pathway between the gut microbiota and the central nervous system, including several humoral, immune and neural routes (Farzi et al., 2018; Dinan and Cryan, 2019). The HPA axis, the stress control system, is a complex network of hormonal interactions between three endocrine glands: the hypothalamus, the pituitary gland, and the adrenal glands (Ulrich-Lai and Herman, 2009). The HPA-axis is highly active in women with distress leading to elevated levels of the hormone cortisol (Nierop et al., 2006a, b; Taylor et al., 2009; Garcia-Leal et al., 2017). Cortisol can directly influence the maternal gut microbiota diversity by altering bile acid production, acidity of the gut secretions, and permeability of the gut, and hence the gut environment for commensal bacteria and gut microbiota diversity (Li et al., 2013; Vanuytsel et al., 2014; Wang and Kasper, 2014; Deloose and Tack, 2016). The potential changes in maternal gut microbiota may have been a result of higher HPA-axis activation in mothers with high psychosocial distress, compared to mothers with low psychosocial distress. In support of this notion, studies among healthy individuals established that self-reported stress was related to reduced diversity of the gut microbiota (Knowles et al., 2008; Kato-Kataoka et al., 2016). Another study, including women of this cohort, found that women with higher psychosocial distress had higher levels of cortisol in milk compared to mothers with low psychosocial distress (Aparicio et al., submitted). Since cortisol passively diffuses from plasma to milk, it is justified to assume that plasma cortisol was higher in mothers with high psychosocial distress than women with low psychosocial distress (Hamosh, 2001). Furthermore, several authors have reported changes in the composition of the fecal microbiota in women with depression, involving shifts in *Actinobacteria* (Chen et al., 2018). Future studies covering not only milk but also maternal gut microbiome analysis may help to gain insight into the link between maternal psychosocial distress and changes in milk microbial composition its impact on infant health.

The lack of significant differences in bacterial relative abundances between women with higher and lower psychological distress, may be due to the fact that this study included a non-clinical population of women. Other authors suggest that alterations in the relative abundance of gut bacteria may only be observable in people with severe psychopathology (Kleiman et al., 2017). To further clarify the relation and underlying mechanisms between psychosocial distress and milk microbiota, future studies may consider examining the gut and milk microbiota of women with a clinical psychopathological status. Additionally, it would be useful to extend the study to a larger population in order to improve the power to detect potential differences between women with high and low psychosocial distress, and to draw definitive conclusions regarding the changes registered in the milk microbial composition of this study.

There are several other factors that, by increasing psychosocial distress, might have contributed to alterations in maternal gut microbiota, and consequently, milk microbial diversity. These factors include motherhood-related social stressors, diet, and sleep. Motherhood-related social stressors, considered as daily circumstances that are perceived problematic or undesirable (such as child care stress, increased infant crying), might have increased psychosocial distress and hence plasma cortisol levels and maternal gut microbiota (Falah-Hassani et al., 2016; Russell and Lincoln, 2016). In fact, one study in rodents found that exposure to social stressors reduced diversity of the gut microbiota (Bailey et al., 2011). Furthermore, poorer sleep quality and a diet low in dietary fiber have been associated with lower gut microbial diversity in non-pregnant population (Graf et al., 2015; Benedict et al., 2016; Barrett et al., 2018). Increased psychosocial distress has been related to poorer sleep quality and disordered eating attitudes (i.e., emotional and restrained eating) among mothers during the first months postpartum (Posmontier, 2008; Emerson et al., 2017). Thus, mothers with high and low psychosocial distress may have had different sleeping patterns and diets, which might have influenced both gut and milk microbiota accordingly.

Previous studies have found that other milk components (e.g., fatty acids, Human milk oligosaccharides (HMOs), lactoferrin, and immune factors) may influence specific bacterial groups in milk (Kim et al., 2004; Ward et al., 2006; LoCascio et al., 2007; Keith et al., 2012; Kumar et al., 2016; Witkowska-Zimny and Kaminska-El-Hassan, 2017; Qi et al., 2018). Lower amounts of fatty acids in milk were related to lower amounts of *Proteobacteria* (Kumar et al., 2016), and HMOs and lactoferrin were found to selectively nourish beneficial *Bifidobacterium* (Kim et al., 2004; Ward et al., 2006; LoCascio et al., 2007). Our study did not examine any of these milk components and thus future research is needed to verify the potential relation between these macronutrients and milk bacteria. Additionally, a previous analysis performed in the same group of women indicated that there was no association between psychosocial distress and the profile of 22 immune factors present in milk samples including innate and acquired immunity factors, chemokines, growth factors and immunoglobulins. Therefore, at this point, it is not reasonable to assume a potential link between immune factors and the microbial diversity in milk (Aparicio et al., submitted).

Higher bacterial diversity of the gut and milk microbiota has been repeatedly associated with improved health status, which raises the question whether the findings on diversity differences in low and high distressed mothers have potential implications for maternal and infant health (Patel et al., 2017; Larsen and Claassen, 2018). For instance, milk of women suffering from mastitis is characterized by a lower microbial diversity (Jiménez et al., 2015; Patel et al., 2017) and a depletion of *Lactobacillus* (Jiménez et al., 2008; Arroyo et al., 2010) and *Acinetobacter* (Patel et al., 2017). For infants, milk microbiota is a main contributor to the infant gut colonization and consequently plays an important role for infant health (Fernández et al., 2013; Bäckhed et al., 2015; Tamburini et al., 2016; Milani et al., 2017). Exposure to increased bacterial diversity in milk may contribute to an increase in bacterial diversity of the infants’ gut microbiota during the first months postpartum (Khodayar-Pardo et al., 2014; Pannaraj et al., 2017). On the contrary, reduced diversity or aberrant composition of milk microbiota may contribute to lower bacterial diversity in the infant gut. Reduced bacterial diversity in early life has been linked to increased risk of gastrointestinal disorders or other diseases (e.g., asthma, metabolic disorders) both during infancy and later, at adult age (Milani et al., 2017). However, the actual impact of the observed changes in the milk microbiota of women having high psychosocial distress remains uncertain because different taxonomic profiles can result in microbiotas with the same functionality due to metabolic plasticity and functional redundancy, similarly to what has been described regarding the infant gut microbiota (Moya and Ferrer, 2016). In order to gain valuable information on the impact of milk microbial composition on the infant health status, it would be desirable to evaluate possible modifications of the functional diversity of milk microbiota as a consequence of the differences in genomic bacterial diversity. If an association between symptoms of anxiety or depression and genomic and functional bacterial milk composition (i.e., relative abundance and diversity) is confirmed, it would be interesting to explore whether maternal probiotic supplementation might restore the milk microbiota (Mastromarino et al., 2015).

The fundamental contribution of this study is that it took first steps to uncover potential relations between maternal psychosocial distress and microbial composition in milk. Another strength of the study is its longitudinal design. Milk is a dynamic biological fluid that changes over the course of lactation, and the longitudinal design of the study enabled us to explore this dynamic process in relation to maternal mood over the first 3 postnatal months. However, there are several limitations to address. The study is inherently limited by the relatively small sample size of women with high and low psychosocial distress, and thus the results of this study should be replicated in larger study populations in order to draw definitive conclusions. Another limitation of this study, which is shared by other metataxonomic studies of samples carrying a low bacterial load such as human milk from healthy women, is related to the high sensitivity of next-generation sequencing. This technique can detect even small amounts of contaminant DNA. Therefore, it is a challenge to exactly characterize microbial communities because DNA from other sources can be introduced in different steps from sampling to final sequencing. For this reason, the interpretation of the metataxonomic studies obtained by high-throughput sequencing technologies of human milk samples should be done with caution (Ruiz et al., 2019). Despite the strict instructions provided to mothers to minimize the risk of external contamination, a small number of samples contained a relatively high content of *Proteobacteria*, which are widely distributed in fecal, soil and water environments. Their presence may indicate either that sampling was occasionally inappropriate (Marín et al., 2009; Jiménez et al., 2017) or the potential presence of contaminating DNA in the molecular biology reagents (Salter et al., 2014). It is important to note, however, that the presence of *Proteobacteria* was unrelated to maternal distress. The main limitation of this study is the oversimplification of the associations investigated. The next step would be to replicate these findings in a large study with additional data and sufficient power to also include analyses of the complex interactions between maternal mood, bodily functioning, environment, and microbial networks in the gut and milk (i.e., microbe-host interactions, microbe-microbe interactions) (Sung et al., 2017). Additionally, due to changes inherently related to the transition to motherhood, these studies should include a questionnaire that measures postnatal stress as a distinct component of psychosocial stress (Hung, 2005; Wu and Hung, 2016), to more precisely tap into the distress that is characteristic of the first postnatal months.

## Conclusion

Although causal effects cannot be identified given the observational nature of the study, the current study is first to establish a potential relation between maternal psychosocial distress and milk bacteria in a population of healthy women after term deliveries. Women with higher levels of psychosocial distress showed a lower bacterial diversity in their milk microbiota at 3 months postpartum as compared to women with lower distress. However, more studies with larger number of participants, and preferably more milk samples, are needed to confirm the potential association between maternal psychosocial distress and milk microbiota. Future research is also necessary to gain further insight into the impact of these changes on the infants’ intestinal microbiome composition, health, and behavior.

## Data Availability Statement

The raw data supporting the conclusions of this manuscript will be made available by the authors, without undue reservation, to any qualified researcher. The sequencing data has been deposited at the European Nucleotide Archive under the project accession number: PRJEB31722.

## Ethics Statement

This study was carried out in accordance with the recommendations of “the Helsinki Declaration (ECG 300107 and ECG 22111/130112)” with written informed consent from all subjects. The protocol was approved by the Ethical Committee of the Faculty of Social Sciences, Radboud University, Nijmegen, Netherlands, provided approval of the study (ECSW2014- 1003- 189).

## Author Contributions

PB prepared the data for analyses and drafted the manuscript. MA performed the experimental part of the project (processing of milk samples), analyzed and interpreted the data, and drafted the manuscript. CA participated in the analysis and the interpretation of the data. CH participated in the design of the study and collected the data. RB designed the study and revised the manuscript. JR designed the study, participated in the funding acquisition, and provided the critical revisions of the manuscript for important intellectual content. LF participated in the design of the study, funding acquisition, analysis and interpretation of the data, and provided a critical revision of the manuscript. CW designed and set up the BINGO study, acquired funding for the BINGO study, and provided the critical revisions of the manuscript for important intellectual content. All authors discussed the results and commented on the manuscript.

## Conflict of Interest

The authors declare that the research was conducted in the absence of any commercial or financial relationships that could be construed as a potential conflict of interest.

## Footnotes

1 https://github.com/jstjohn/SeqPrep

2 https://www.R-project.org
