# Supplementary material for: Human Milk Microbiome and Maternal Postnatal Psychosocial Distress
Source: Front Microbiol. 2019 Oct 22;10:2333. doi: 10.3389/fmicb.2019.02333 (PMC6817470; doi:10.3389/fmicb.2019.02333)
Supplement: Supplementary file 1 [file Data_Sheet_1.pdf]

Supplementary Material

Supplementary Figures

Supplementary Figure 1. Flowchart of the included participants in the study.

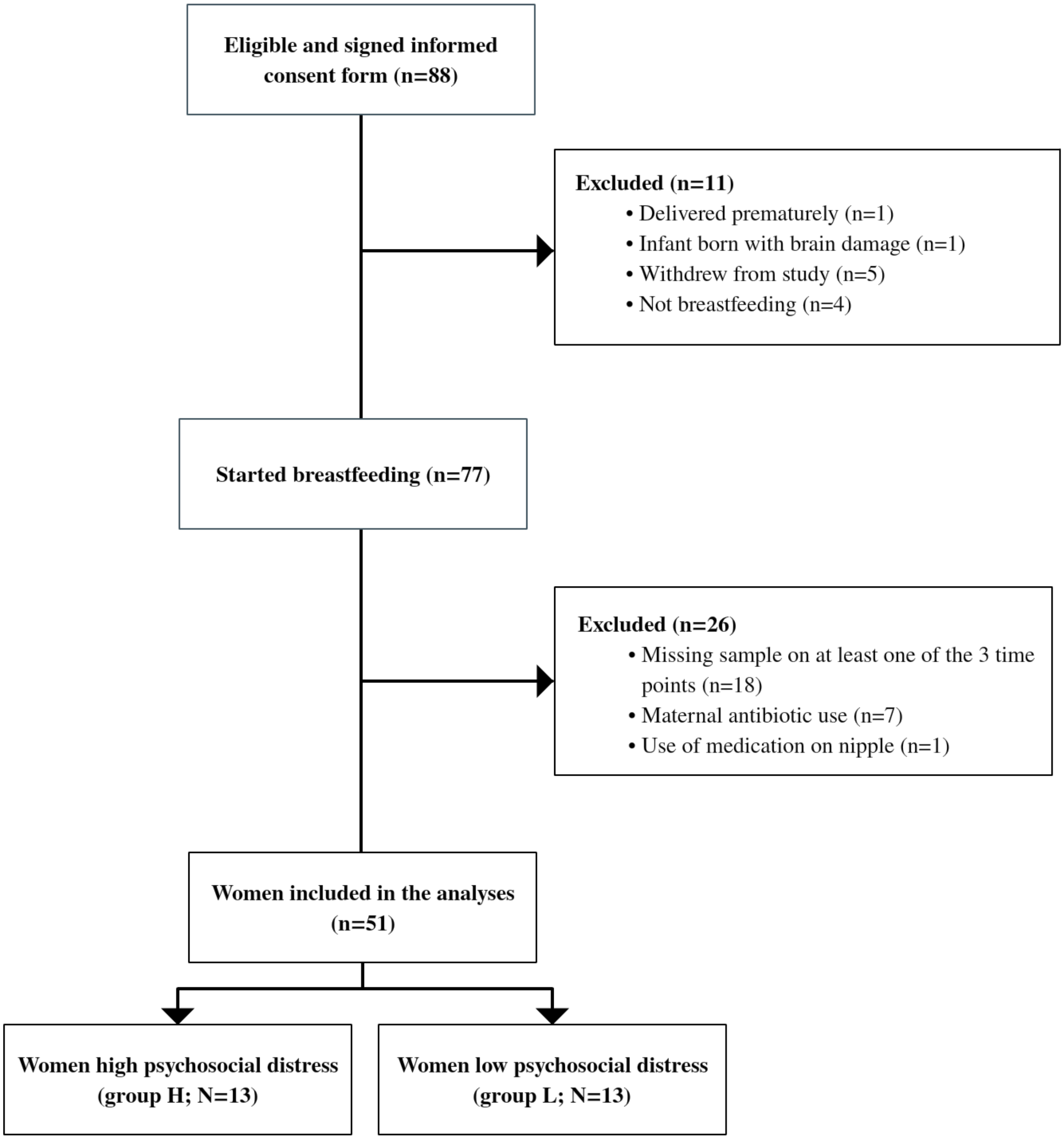

Supplementary Figure 2. Rarefaction curves of OTUs per sequences of 152 milk samples.

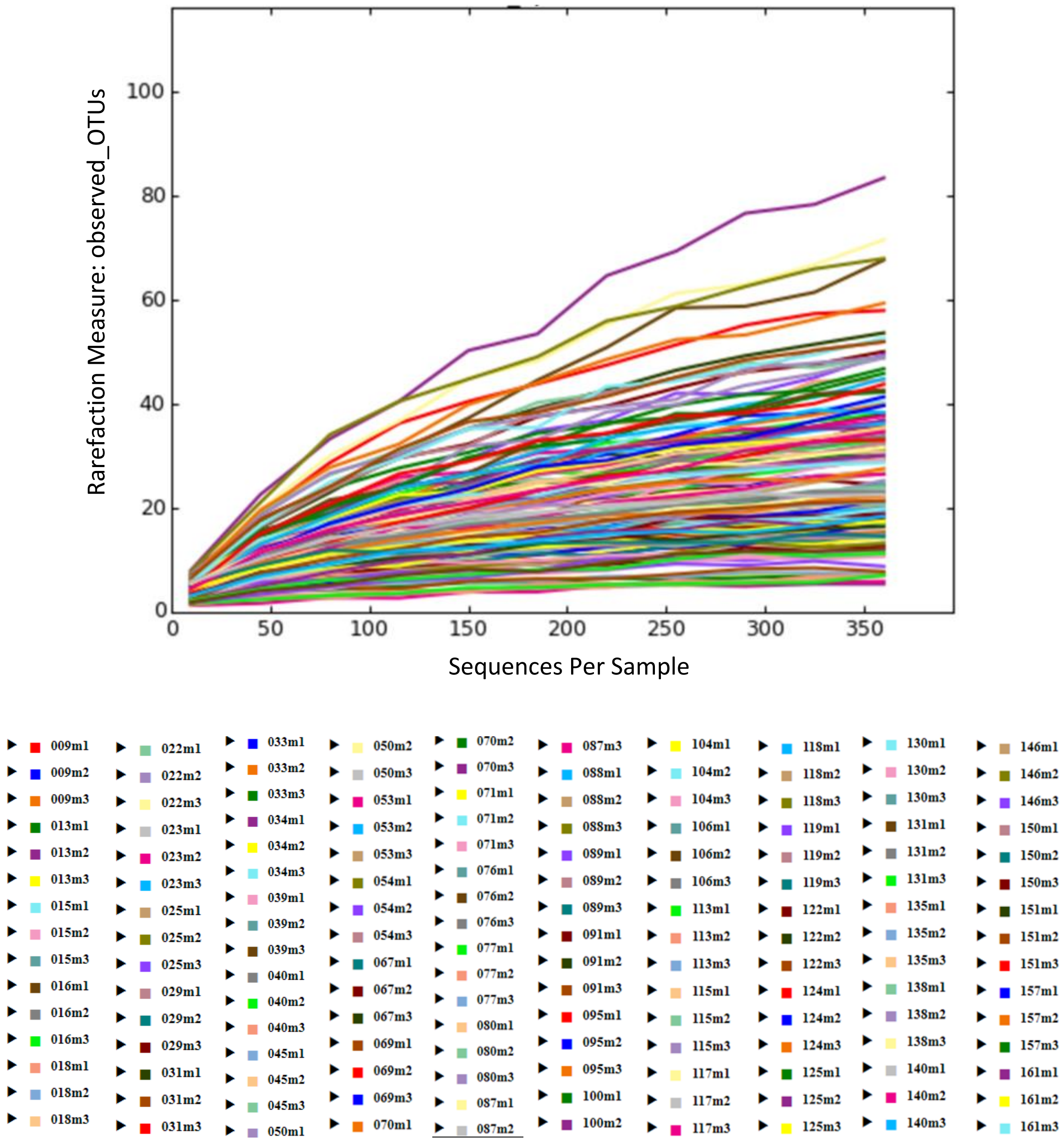

Supplementary Material

Supplementary Tables

Supplementary Table S1. Relative abundance of operational taxonomic units at domain level in human milk samples (n=152).

Domain	Mean	Median	IQR	min	max
Bacteria (n=152)	97.38	98.20	96.67-99.21	69.06	99.76
Archaea* (n=20)	0.17	0.12	0.08-0.21	0.03	0.67
Unclassified (n=152)	2.60	1.78	0.79-3.30	0.24	30.94

*Mean, median, and IQR values correspond to these 20 samples.

Supplementary Table S2. Presence and relative abundance (% of total) of operational taxonomic units at phylum level in all milk samples at weeks 2, 6 and 12 after delivery.

	Week 2 (n=51)		Week 6 (n=51)		Week 12 (n=50)			
**Phylum**	**n (%)**	**Median (IQR) (% of total OTUs)**	**n (%)**	**Median (IQR) (% of total OTUs)**	**n (%)**	**Median (IQR) (% of total OTUs)**	***p*-value#**	***p*-valueǂ**
Firmicutes	51 (100%)	86.51 (68.03–93.63)a	51 (100%)	74.54 (52.71– 1.09)ab	50 (100%)	58.51 (39.22–86.27)b	-	0.018
Proteobacteria	50 (98%)	7.87 (2.60–20.34)a	51 (100%)	12.35 (4.34–26.94)ab	50 (100%)	21.79 (5.96–45.67)b	-	0.023
Actinobacteria	50 (98%)	0.87 (0.34–.62)	48 (94%)	1.02 (0.38–1.95)	45 (90%)	1.07 (0.50–2.21)	0.173	0.820
Bacteroidetes	45 (88%)	1.18 (0.37–3.51)a	47 (92%)	1.30 (0.52–5.39)ab	50 (100%)	1.14 (0.38–8.61)b	0.036	0.049
Unclassified	51 (100%)	1.87 (0.83–2.92)	51 (100%)	1.80 (0.73–3.38)	50 (100%)	1.73 (0.91–3.59)	-	0.383
Minor phyla*(<90%)	40 (78%)	0.45 (0.23–0.84)	43 (84%)	0.60 (0.26–1.45)	43 (86%)	0.72 (0.27–2.21)	0.635	0.059

#Chi-squared or Fisher exact probability tests were used to evaluate differences in expression frequencies of the analyzed parameters across time.
ǂFriedman tests were used to evaluate differences in relative abundance along time. Different caption letters mean statistical differences when the *post hoc* pairwise comparison Nemenyi test was done.
*Minor phyla groups all phyla present in <90% of the total samples.

Supplementary Table S3. Presence and relative abundance (% of total) of operational taxonomic units at genus level in all milk samples at weeks 2, 6 and 12 after delivery.

	**Week 2 (n=51)**		**Week 6 (n=51)**		**Week 12 (n=50)**			
**Genus**	**n (%)**	**Median (IQR)**	**n (%)**	**Median (IQR)**	**n (%)**	**Median (IQR)**	***p*-value#**	***p*-value‡**
Staphylococcus	51 (100%)	58.71 (35.33–79.60)a	51 (100%)	44.65 (21.99–66.71)ab	50 (100%)	24.81 (10.16–59.32)b	-	0.001
Streptococcus	51 (100%)	8.02 (2.50 –20.94)	49 (96%)	10.72 (2.63–27.46)	47 (94%)	7.46 (1.94–20.31)	-	0.478
Corynebacterium	41 (80%)	0.21 (0.08–0.35)	40 (78%)	0.14 (0.06–0.30)	36 (72%)	0.20 (0.10–0.36)	0.577	0.397
Gemella	39 (76%)	3.20 (0.55–6.56)	41 (80%)	1.49 (0.57–4.05)	36 (72%)	0.61 (0.24–2.97)	0.613	0.389
Acinetobacter	36 (71%)	0.69 (0.17–6.13)	37 (73%)	0.93 (0.35 –3.47)	39 (78%)	0.77 (0.23–19.62)	0.680	0.446
Haemophilus	35 (69%)	0.29 (0.09–0.67)	29 (57%)	0.46 (0.13–1.29)	30 (60%)	0.47 (0.13–1.16)	0.449	0.771
Pseudomonas	35 (69%)	0.28 (0.14–0.85)	36 (71%)	0.55 (0.30–1.21)	34 (68%)	0.47 (0.11 –1.84)	0.956	0.792
Propionibacterium	34 (67%)	0.27 (0.08–0.71)	35 (69%)	0.23 (0.15–0.44)	37 (74%)	0.28 (0.12–0.50)	0.708	0.811
Lactobacillus	34 (67%)	0.37 (0.19–1.21)a	39 (76%)	0.79 (0.38–2.07)ab	39 (78%)	1.35 (0.25–2.53)b	0.372	0.007
Veillonella	33 (65%)	0.21 (0.09–0.65)	33 (65%)	0.37 (0.17–0.70)	27 (54%)	0.28 (0.11–0.53)	0.445	0.173
Enhydrobacter	31 (61%)	0.32 (0.14–0.88)	36 (71%)	0.33 (0.14–1.03)	34 (68%)	0.58 (0.15–1.03)	0.554	0.157
Flavobacterium	28 (55%)	1.72 (0.36–3.79)	32 (63%)	1.23 (0.33–4.74)	33 (66%)	2.49 (0.41–8.52)	0.499	0.240
Minor genera[a]	51 (100%)	9.46 (5.25–20.49)a	51 (100%)	17.35 (7.15–30.16)b	50 (100%)	19.38 (9.00–37.26)b	-	0.021
Unclassified	51 (100%)	5.26 (3.31–8.97)	51 (100%)	7.86 (3.58–11.14)	50 (100%)	7.44 (4.04–14.43)	-	0.382

#Chi-squared tests were used to evaluate differences in detection frequencies of the analyzed bacterial genera across time.
‡Friedman tests were used to evaluate differences in relative abundance along time. Different caption letters mean statistical differences when the *post hoc* pairwise comparison Nemenyi test was done.
[a]Minor genera includes bacterial genera with a relative abundance of <0.1%.

Supplementary Table S4. Change in diversity indices of operational taxonomic units in all milk samples at weeks 2, 6 and 12 after delivery.

	**Week 2 (n=51)**		**Week 6 (n=51)**		**Week 12 (n=50)**		
	**Median (IQR)<br>Mean (95% CI)**	**Range<br>(min, max)**	**Median (IQR)<br>Mean (95% CI)**	**Range<br>(min, max)**	**Median (IQR)<br>Mean (95% CI)**	**Range<br>(min, max)**	***p*-value**
Observed OTUs[a]	38 (26–49)	(10, 163)	42 (29–59)	(9, 138)	46 (32–65)	(11, 127)	0.058
Shannon[b]	2.38 (2.18, 2.58)a	(0.25, 4.84)	2.54 (2.34, 2.74)ab	(0.36, 4.30)	2.68 (2.48, 2.89)b	(0.57, 5.00)	0.006
Simpson[a]	0.65 (0.52–0.79)	(0.05, 0.92)	0.69 (0.52–0.80)	(0.08, 0.90)	0.72 (0.57–0.84)	(0.14, 0.94)	0.923

[a] Data for observed OTUs and Simpson index are expressed as medians (IQR), and the differences for observed OTUs and Simpson index along time were evaluated using Friedman tests.

[b] Data for Shannon index are expressed as means (95% CI), and the differences along time were evaluated using ANOVA for repeated measures. Different caption letters mean statistical differences between pairs of values using the Bonferroni correction for multiple comparison.
